# Functional analysis of hemichannels and gap-junctional channels formed by connexins 43 and 46

**Published:** 2010-07-15

**Authors:** Quan V. Hoang, Haohua Qian, Harris Ripps

**Affiliations:** 1 Departments of Ophthalmology and Visual Sciences, University of Illinois at Chicago, Chicago, IL; 2 Biological Sciences, University of Illinois at Chicago, Chicago, IL; 3 Anatomy and Cell Biology, University of Illinois at Chicago, Chicago, IL; 4 The Marine Biological Laboratory, Woods Hole, MA

## Abstract

**Purpose:**

The gap junctions (GJs) mediating direct cell-cell interaction are formed by clusters of membrane-spanning proteins known as connexins (Cxs). These channels play a key role in signal transmission, and their permeability, time-, and voltage-dependence are governed by the properties of the specific Cxs forming the gap junctions. Retinal pigment epithelium (RPE) cells express Cx43 and Cx46. Here, we employed a heterologous expression system to explore the functional properties of the hemichannels and GJs that could be formed by different combinations of these Cxs. Specifically, we examined the response kinetics of GJs formed by pairing cells expressing Cx43 or Cx46, or those expressing both, i.e., designated as Cx43•Cx46.

**Methods:**

The *Xenopus* oocyte expression system and a two-electrode voltage clamp technique were used to study the properties of hemichannels and GJs formed in oocytes transfected with Cx43 and/or Cx46 mRNA.

**Results:**

Depolarizing voltages activated hemicurrents of similar amplitude from single oocytes transfected with Cx46 or Cx43•Cx46, but not in oocytes expressing Cx43 alone. Incorporating Cx43 with Cx46 altered the gating charge, but not the voltage sensitivity of the hemichannels. In addition, Cx43•Cx46 hemichannel currents exhibited faster activation kinetics than homomeric Cx46 hemichannels. Both homotypic GJs formed by Cx43 and Cx46, and heteromeric Cx43•Cx46 GJs exhibited large junctional conductances with amplitudes of 6.5±3.0 μS (Cx43), 8.9±3.4 μS (Cx46), and 8.5±1.8 μS (Cx43•46); a significantly lower conductance (1.8±0.7 μS) was observed for heterotypic GJs formed by Cx43 and Cx46. There were also differences in their gating kinetics. Whereas the kinetics of homotypic Cx46 could be described by a single exponential function (τ=0.91 s), double exponential functions were required for homotypic Cx43 (τ_1_=0.24, τ_2_=3.4 s), heterotypic Cx43/Cx46 (τ_1_=0.29, τ_2_=3.6 s), and heteromeric Cx43•Cx46/Cx43•Cx46 (τ_1_=1.2, τ_2_=8.1 s) junctions.

**Conclusions:**

The failure of oocytes expressing Cx43 to exhibit hemichannel activity is an intrinsic membrane property of this Cx, and cannot be attributed to a lack of expression; western blot analysis showed clearly that Cx43 was expressed in oocytes in which it was injected. Our results provide further evidence that Cx43 and Cx46 form both heterotypic and heteromeric channels when co-expressed, an indication that various combinations of Cxs may participate in gap-junctional communication between RPE cells.

## Introduction

Gap junctional channels are found in tissues throughout the body and play a key role in signal transmission and other cellular processes by allowing for direct cell-cell communication, i.e., the intercellular exchange of ions, metabolites, and small peptides having a molecular mass of ≤1 kDa [1-4]. The building blocks of the gap-junctional channel are connexins (Cxs), a family of homologous transmembrane proteins whose members are distinguished based on their predicted molecular mass in kDa, e.g., Cx25, Cx31, and Cx45. Six Cx polypeptides oligomerize to form a hemichannel or connexon, which docks with a connexon from an adjacent cell to create an aqueous pore that bridges the ~2 nm intercellular “gap.” Variation in Cx assembly can lead to multiple gap-junctional configurations exhibiting very different communication properties [5]. *Homotypic* gap junctions are typically formed between coupled cells that contain one type of Cx. If each of the paired cells expresses a different Cx, a *heterotypic* gap junction can result. However, when the connexons of each cell consist of more than one type of Cx, they may form *heteromeric* gap junctions [1,6-9].

Dye coupling and electrophysiological studies have shown that all retinal cells communicate with their neighbors via gap junctions (GJs) [10-13]. In retinal pigment epithelial (RPE) cells, the Cxs mediating gap-junctional intercellular communication have been identified as Cx43 and Cx46 using immunocytochemical [14] and immunoblot analysis [15]. Therefore, all three types of GJs could exist, i.e., homotypic (Cx43/Cx43) or (Cx46/Cx46), heterotypic (Cx43/Cx46), or heteromeric (where both hemichannels are composed of a mixture of Cx43 and 46 [Cx43•46/Cx43•46]). Indeed, there is evidence that these proteins form heterotypic channels with novel properties not predicted by their homotypic counterparts [16]. Moreover, it has been demonstrated that Cx43 and Cx46 are capable of forming heteromeric complexes when expressed in HeLa and alveolar epithelial cells [17,18]. In the present study, we employed a heterologous system (*Xenopus* oocytes) to explore the electrophysiological properties of the hemichannels and GJs formed by the Cxs normally expressed in RPE cells, and provide additional evidence that Cx43 and Cx46 form heteromeric channels when co-expressed.

## Methods

### Ethics statement

All experimental procedures were performed in accordance with the National Institute of Health Guide for the Care and Use of Laboratory Animals (NIH Publications No. 80–23), revised in 1996, and adhered to the guidelines for the care and use of laboratory animals formulated by the Association for Research in Ophthalmology and Visual Sciences (ARVO). All procedures were approved by the Animal Care Committee of the University of Illinois at the Chicago College of Medicine. Oocytes of the South African clawed toad *Xenopus laevis* are widely used as a system for expression and characterization of exogenous membrane channels. The cells provide a convenient test environment, are large and easily penetrated for microinjection and recording, and have been used extensively for heterologous expression of ion channels, neurotransmitter receptors, transporters, and other membrane proteins.

### Oocyte preparation

The procedures for oocyte preparation and Cx expression were described previously [19]. Briefly, ovarian lobes were removed under surgical anesthesia (0.02% 3-aminobenzoic acid ethyl ester, Tricaine) from gravid *Xenopus laevis* females (*Xenopus* I, Dexter, MI), and incubated with constant agitation for 2 h in a calcium-free modified Barth’s (MB) solution containing collagenase (2.5 mg/ml), gently triturated, and rinsed twice in Ca^2+^-free MB without collagenase. Defolliculated stage V-VI oocytes were selected and repeatedly rinsed in MB solution containing (in mM): NaCl (88), KCl (1), NaHCO_3_ (2.4), HEPES (N-2-hydroxyethylpiperazine-N’-2-ethaneesulfonic acid) (15), Ca(NO_3_)_2_ (0.33), CaCl_2_ (0.41), and MgSO_4_ (0.82); 10 mg/l gentamycin (Gibco/BRL) was added, and the solution was titrated with NaOH to pH 7.6.

Plasmids containing the coding sequences of mouse Cx43 and mouse Cx46, generously provided by Dr. Thomas White (SUNY, Stony Brook, NY), were subcloned into the BamH1 site of the pCS2+ expression vector. The constructs were linearized with the NotI restriction endonuclease, and capped mRNAs were transcribed in vitro with SP6 RNA polymerase using the mMessage mMachine (Ambion Inc., Austin, TX). The oocytes were maintained in MB at 15 °C for 2 h to two days before injection of cRNAs into the vegetal poles of the oocytes using a Nanoject Injector (Drummond Scientific Co., Broomall, PA). The injection pipettes were drawn on a vertical electrode puller (Narishige PB-7, Tokyo, Japan), and broken under microscopic observation to a tip opening of 10–15 μm. Oocytes were injected with 46 nl of an aqueous solution containing 13 ng/cell of Cx43 and/or 13 ng/cell of Cx46, together with 10 ng/cell of an antisense oligomer to nucleotides 128–151 of the coding region of the endogenously-expressed Cx38. For control experiments, oocytes were injected only with a solution containing 10 ng/cell of the antisense oligomer.

### Electrophysiology

Nonjunctional (hemichannel) currents were recorded ~2 days after transfection from individual oocytes mounted on a 0.5 mm nylon mesh in a specially constructed lucite chamber (volume=0.7 ml) using a two-electrode voltage clamp [20]. Cells were clamped at −20 mV, and whole cell currents were recorded in response to depolarizing voltage steps (−10 to +60 mV at 10 mV intervals) imposed for a duration of 10 s. Current values measured at the end of the pulse were plotted against membrane potential. In experiments designed to determine gap junctional conductances, the vitelline envelopes of the oocytes were removed in a hypertonic solution one day after transfection, and the cells manually paired with vegetal poles apposed in Petri dishes containing MB supplemented with 5% Ficoll. For both types of experiments, current and voltage electrodes were pulled to resistances of 0.7–1.5 MΩ when filled with an internal solution of 3 M KCl, 10 mM EGTA, and 10 mM HEPES, at pH 7.4. The external solution was MB with 5% Ficoll.

Voltage gating and related properties of the intercellular channels between oocytes were measured ~two days after transfection, and one day after oocyte pairing using a dual-cell voltage-clamp technique. To quantitate junctional conductance (G_j_), the two cells of a pair were clamped at −40 mV, close to their initial resting potential, and transjunctional current measurements were obtained in response to 20 mV depolarizing steps applied alternately to each of the cells. Under these conditions, the current applied by the clamp to the cell not stepped is equal in amplitude, but opposite in sign to the junctional current (I_j_). Junctional conductance was then calculated by dividing the junctional current by the transjunctional potential. To determine the voltage-gating properties of the intercellular channels, transjunctional potentials of opposite polarity were generated by hyperpolarizing or depolarizing one cell in 20 mV steps (over a range of ±120 mV), while clamping the second cell at −40 mV. After each voltage step, steady-state currents (I_jss_) were measured 10 s after the onset of the voltage pulse. Steady-state conductances (G_ss_) were normalized to their value at ±20 mV, and plotted against the transjunctional potential. Voltage clamping of paired oocytes was performed using two GeneClamp 500B amplifiers controlled by pClamp 8 software through a Digidata 1322A interface (Axon Instruments, Molecular Devices, Sunnyvale, CA). Hemichannel currents and GJIC currents were analyzed using the pClamp 8 software suite (Axon Instruments).

**Figure 1:**
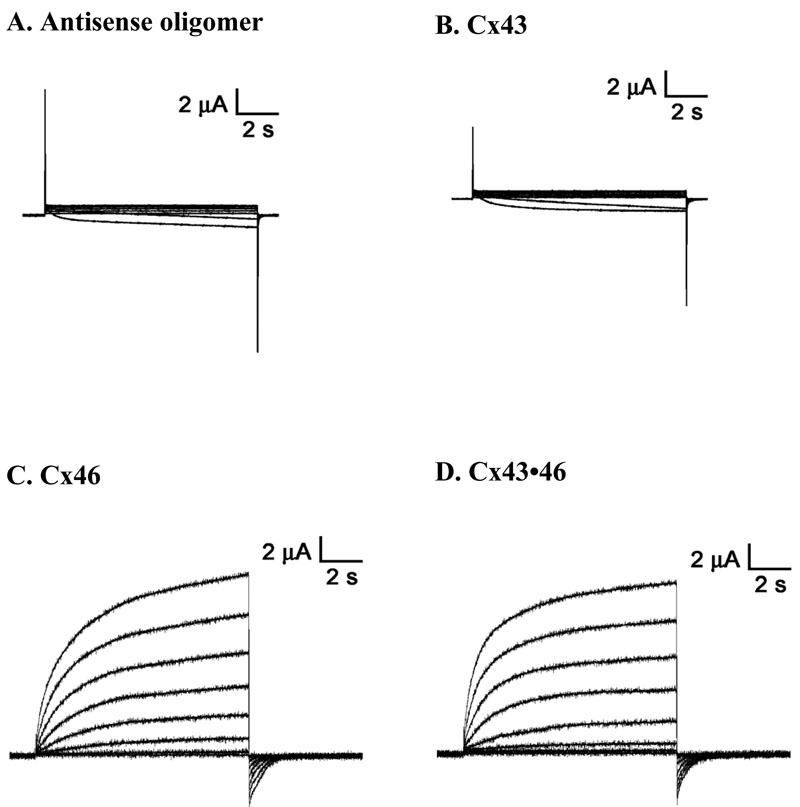
Membrane currents recorded from single cells in response to depolarizing voltage steps from a holding potential of −20 mV, and stepped in 10 mV increments from −10 mV to +60 mV. The oocytes were injected with 46 nl of an aqueous solution containing an antisense nucleotide to the endogenous Cx (Cx38) normally expressed by *Xenopus* oocytes, either alone (**A**) or along with (**B**) 13 ng of the cRNA encoding Cx43, (**C**) a similar concentration of Cx46, and (**D**) a mixture containing 13 ng each of Cx43 and Cx46. Note that the outward currents typical of hemichannel activity were absent from oocytes receiving either the antisense oligomer or Cx43.

Data describing the macroscopic junctional conductance as a function of transjunctional voltage were analyzed using Microcal Origin 5.0 software (Microcal Software Inc., Northampton, MA) and fit to a Boltzmann relation [21] of the form:

G_jss_={(G_jmax_ – G_jmin_)/(1 + exp[A (V_j_ – V_o_)])} + G_jmin_ (1)

where G_jss_ is the steady-state junctional conductance, G_jmax_ (normalized to unity) is the maximum conductance, G_jmin_ is the residual conductance at large values of junctional voltage (V_j_), and V_o_ is the transjunctional voltage at which G_jss_=(G_jmax_ – G_jmin_)/2. The constant A (=*nq/kT*) represents the voltage sensitivity in terms of gating charge as the equivalent number (*n*) of electron charges (*q*) move through the membrane, *k* is the Boltzmann constant, and *T* is the absolute temperature. The time constants (τ) of voltage-dependent transitions of junctional conductance were calculated using data fitting functions in Microcal Origin.

### Western-blot analysis

Oocytes were harvested two days after transfection with Cx mRNA. Non-expressing oocytes served as controls. The membrane protein fraction of the oocytes was prepared using the method described previously [22]. Next, 50 μg of total membrane proteins were loaded in each lane and separated on SDS–PAGE. After transfer to a polyvinylidene difluoride membrane, the blot was probed with primary antibody (1:500 dilutions; Invitrogen) overnight at 4 °C, followed by 1 h incubation with horseradish peroxidase-conjugated secondary antibody. The signals on the blot were detected using SuperSignal® West Femto Maximum Sensitivity Substrate (Pierce, Rockford, IL).

### Statistical analysis

Data analysis tools in Microsoft Excel were used to calculate statistical significance (two-tail *t*-test analysis).

## Results

### Hemichannel currents

**Figure 2:**
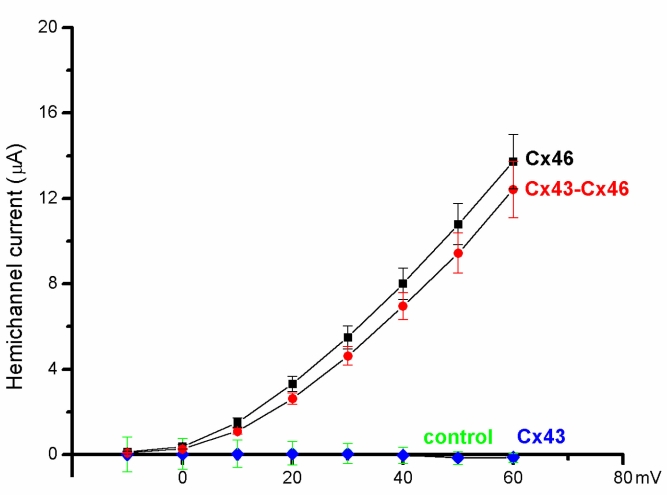
Current-voltage relationships (averaged data) for cells expressing Cx46 (n=9) and the combined cRNA derived from the mixture of Cx43 and Cx46 (n=11). The curves for the antisense (control, n=10) and Cx43 (n=12) injected cells remained at baseline over the entire voltage range.

Figure 1 illustrates examples of membrane currents elicited by voltage pulses from individual *Xenopus* oocytes injected with antisense Cx38 oligonucleotide alone or expressing Cx43, Cx46, or Cx43 with Cx46 (Cx43•46). In oocytes injected with the antisense Cx38 nucleotide, minimal membrane currents were observed in response to voltage steps from −10 mV to +60 mV (Figure 1A). This finding is similar to the results obtained by Ebihara [23], who reported that large hemichannel currents (>100 nA at +30 mV) are elicited from oocytes not injected with the antisense oligomer, whereas after injection the current is close to zero. As shown in Figure 1A and Figure 2, the antisense oligomer had fully blocked any hemichannel activity that might be attributed to Cx38. Interestingly, the recordings obtained from oocytes expressing Cx43 did not differ from those with the antisense nucleotide, i.e., there was no evidence of significant hemichannel activity (Figure 1B). This is consistent with observations reported in several studies showing that Cx43 hemichannels have a very low open probability under normal conditions in those tissues in which they are expressed [24]. However, they can be opened, e.g., in cardiac myocytes, in response to mechanical, osmotic, and oxidative stress or changes in extracellular or intracellular Ca^2+^, whereupon they release small ions and molecules with signaling potential such as ATP [25]. On the other hand, when expressed in oocytes, the hemichannel conductance is so low as to be undetectable (Figure 1B and Figure 2), a finding first reported by White et al. [26]. In contrast, voltage-activated outward hemicurrents were obtained from cells transfected with Cx46 in response to depolarizing voltage steps ≥+10 mV (Figure 1C), and similar current responses were observed in oocytes co-expressing Cx46 with Cx43 (Figure 1D).

Figure 2 illustrates the mean voltage-activated currents, averaged from several cells, for the various Cx hemichannel configurations. Note that responses of similar magnitude were obtained from oocytes expressing Cx46 and Cx43•46 over the entire range of voltages tested, and in response to a +60 mV voltage pulse, the hemichannel currents were 13.7±1.3 µA for Cx46 (mean±SEM, n=9), and 12.4±1.3 µA for Cx43•Cx46 (mean±SEM, n=11). In contrast, oocytes expressing Cx43 (n=12) or the antisense oligo (n=10) gave extremely small inward currents at this voltage that did not differ significantly from baseline. In a few instances, larger inward currents were seen, probably due to the endogenous sodium channels present in these cells [27].

**Figure 3:**
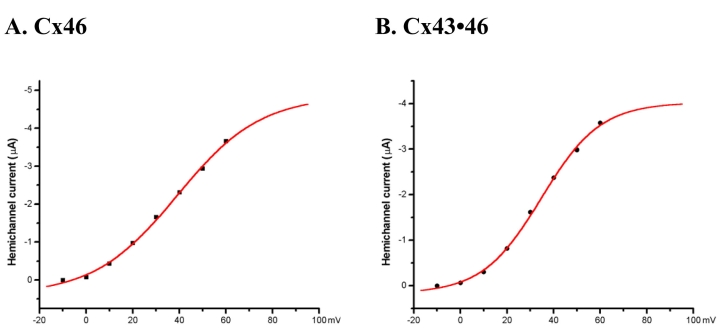
The voltage activation profile examined by tail current analysis in hemichannels from representative cells expressing (**A**) Cx46 or (**B**) the combined cRNA derived from the mixture of Cx43 and Cx46 (Cx43**•**Cx46). The amplitude of the tail current was derived by extrapolating back to the end of the 10 s voltage step. Continuous lines represent curves fit with the Boltzmann equation. Although of similar shape, the curve describing the hemicurrent recorded from oocytes expressing Cx43•46 has a steeper slope, which reflects the difference in gating charge.

The voltage activation profile of the hemichannels expressed by Cx46 and Cx43•46 was examined using tail current analysis. The amplitude of the tail current was derived by extrapolating back to the end of the 10 s voltage step using data fitting functions in the pClamp 8 software suite. Examples of the voltage activation profile for hemichannels formed in oocyte membranes by Cx46 and Cx43•46 are shown in Figure 3; the continuous lines represent curves fit with the Boltzmann equation. Although of similar shape, the curve describing the hemicurrent recorded from oocytes expressing Cx43•46 has a steeper slope, which reflects the difference in gating charge (*n*) in the Boltzmann equation.

Table 1 presents the averaged values for the parameters used in fitting the Boltzmann equation. Notably, the gating charge (*n*) for Cx46 was found to be 1.4±0.05 (mean±SEM, n=9) and it was 1.6±0.07 (mean±SEM, n=11) for Cx43•46; the difference is statistically significant with a p-value of 0.01 (two-tailed *t*-test). These results indicate that incorporating Cx43 with Cx46 alters the voltage sensitivity of the hemichannels expressed in the oocyte membrane. On the other hand, values of V_o_, the membrane voltage at which half of the channels are activated, are similar for cells expressing Cx46 or Cx43•46, i.e., 39.3 mV for Cx46 and 39.5 mV for Cx43•46 (Table 1).

**Table 1:** Boltzmann parameters for hemichannels composed of mCx46 or mCx43 and mCx46.

**Channel**	**A**	**n**	**Vo**
Cx46	0.05	1.4	39.3
Cx43/46	0.06	1.6	39.5
p value	p=0.08	p=0.01	p=0.95

**Figure 4:**
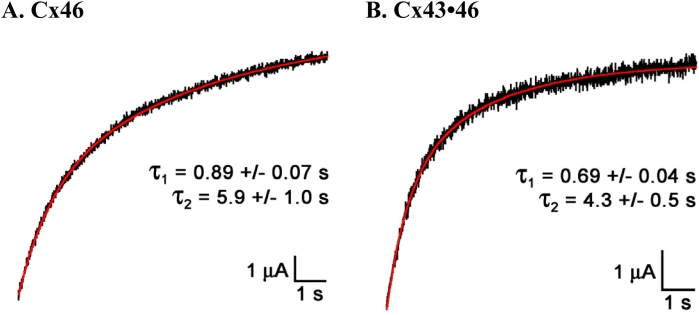
The kinetics of hemichannel activation determined from the response to a +40 mV voltage step in representative cells expressing (**A**) Cx46 or (**B**) the combined cRNA derived from the mixture of Cx43 and Cx46 (Cx43•Cx46). For both types of hemichannels, the activation time course was best fit with a two-exponential function. The mean activation time constants (τ) are shown for Cx46 (n=9) and Cx43•46 (n=12). Note that Cx43•46 hemichannel currents exhibit faster activation kinetics than homomeric Cx46 hemichannels.

The kinetics of hemichannel activation were examined for voltage steps to +40 mV, a potential close to V_o_ for both Cx46 and Cx43•46. For both hemichannels, the activation time course was best fit with a two-exponential function (examples shown in Figure 4). The mean activation time constants (τ) were 0.89±0.07 s and 5.9±1.0 s (n=9) for oocytes expressing Cx46, and 0.69±0.04 s and 4.3±0.5 s (n=12) for cells expressing Cx43•46. In other words, Cx43•46 hemichannel currents exhibit faster activation kinetics than homomeric Cx46 hemichannels. These differences are statistically significant for the fast phase of the time constant (p=0.017, two-tailed *t*-test), but not for the slow phase (p=0.14). In addition, the proportion of fast and slow components in the hemichannel currents is also statistically insignificant for cells expressing Cx46 alone and those co-expressing Cx43 and Cx46 (p=0.4).

### Gap junctional conductance

**Figure 5:**
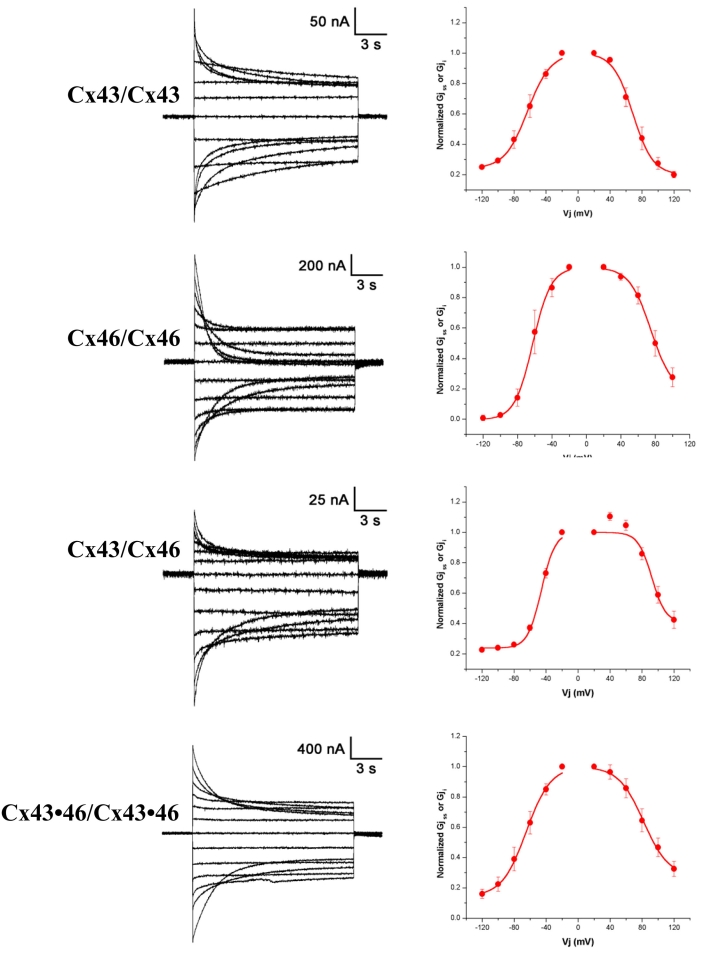
Junctional current (I_j_) data from paired oocytes. The left column shows examples of current traces recorded from homotypic pairs (Cx43/Cx43 and Cx46/Cx46), a heterotypic pair (Cx43/Cx46), and a heteromeric pair (Cx43•46/Cx43•46). The right column plots normalized steady-state junctional conductances (filled circles) measured at the end of 10-s-long voltage steps, and fit to the Boltzmann equation (curved lines).

Figure 5 illustrates junctional current (I_j_) data from paired oocytes. The left column in Figure 5 shows examples of current traces recorded from homotypic pairs (Cx43/Cx43 and Cx46/Cx46), a heterotypic pair (Cx43/Cx46), and a heteromeric pair (Cx43•46/Cx43•46). The right column in Figure 5 plots normalized steady-state junctional conductances (filled circles) measured at the end of 10-s-long voltage steps, and fit to the Boltzmann equation (curved lines). The corresponding Boltzmann parameters for positive and negative values of V_j_, including the gating charge *n*, are given in Table 2.

**Table 2:** Boltzmann parameters for gap-junctional channels composed of mCx43, mCx46, and the two connexins expressed in each of the paired cells.

**Channel**	**V_j_**	**A**	***n***	**V_o_**	**G_jmin_**
Cx43/Cx43	(+)	0.08	2	69	0.21
	(-)	0.07	1.8	63	0.24
Cx46/Cx46	(+)	0.08	2.1	75	0.18
	(-)	0.09	2.4	62	0
Cx43/Cx46	(+)	0.12	2.9	92	0.4
	(-)	0.12	3	46	0.23
Cx43•46/Cx43•46	(+)	0.06	1.6	82	0.28
	(-)	0.07	1.7	65	0.14

For both homotypic pairs formed in oocytes, large junctional conductances (G_j_) were observed, with values of 6.5±3.0 μS (Cx43, n=5) and 8.9±3.4 μS (Cx46, n=3; Table 3). On the other hand, the junctional conductance of the heterotypic channels formed by Cx43 and Cx46 was significantly less (1.8±0.7 μS, n=6). In addition, a large asymmetry can be seen for heterotypic junctional channels, with voltage sensitivities of 92 mV and 46 mV, and G_jmin_ of 0.40 and 0.23 at +V_j_ and –V_j_, respectively. A large junctional conductance was also observed in paired oocytes expressing both Cx43 and Cx46 (8.5±1.8 μS, n=10). The pattern of voltage sensitivity for the junctional current in these cells is more similar to homotypic pairs than the asymmetry displayed by heterotypic pairs.

**Table 3:** mCx43 and mCx46 form homotypic, heterotypic, and heteromeric intercellular channels.

**RNA injection (cell 1/cell2)**	**Junctional conductance in μS (number of pairs)**
Cx43/Cx43	6.5±3
	5
Cx46/Cx46	8.9±3.4
	3
Cx43/Cx46	1.8±0.7 (p=0.05)
	6
Cx43•Cx46/Cx43•Cx46	8.5±1.8
	10

**Figure 6:**
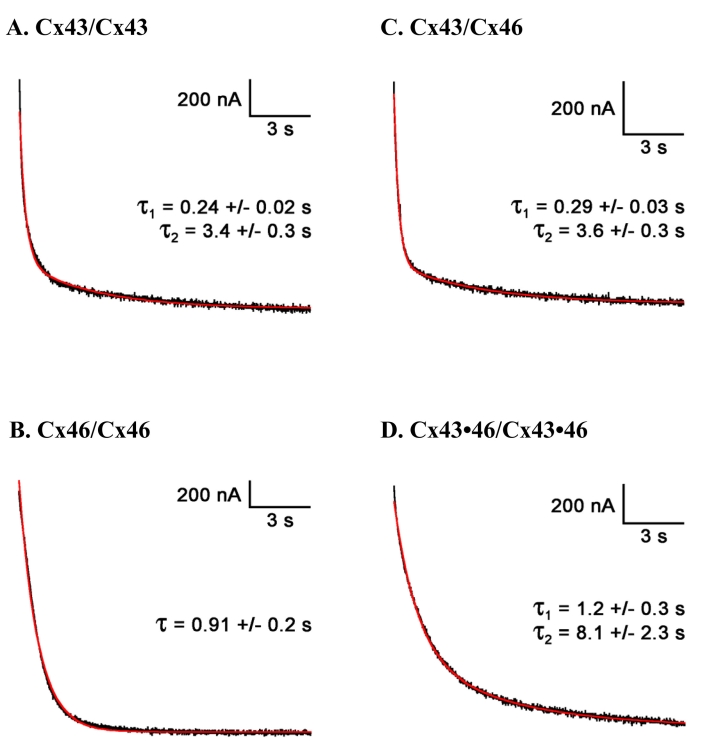
The kinetics of the voltage-dependent closure of gap junctional channels for homotypic (**A**, **B**), heterotypic (**C**), and heteromeric (**D**) pairs of RPE Cxs expressed in *Xenopus* oocytes. For homotypic pairs of Cx43/Cx43 (**A**), the junctional current was best fit with a second-order exponential decay, whereas homotypic Cx46 (**B**) could be described by a single exponential function with averaged time constants shown above (n=5 and n=3, respectively). Gating kinetics for the heterotypic Cx43/Cx46 gap junctional channel (**C**) also required two exponential components (n=6). Heteromeric gap junctions (Cx43•46/Cx43•46) exhibited significantly slower gating kinetics, requiring a second-order exponential function, with the time constants shown in **D**, above (n=10).

The gating kinetics of the gap junctional channels were derived from the responses to –120 mV voltage pulses. Figure 6 shows examples of junctional current traces recorded from each of the junctional pairs investigated in this study. For homotypic pairs of Cx43/Cx43, the junctional current was best fit with a second-order exponential decay, with averaged time constants of 0.24±0.02 s and 3.4±0.3 s (n=5). Interestingly, the kinetics of the voltage-dependent closure of gap junctional channels formed by homotypic Cx46 could be described by a single exponential function with a mean time constant of 0.91±0.02 s (n=3). As the heterotypic Cx43/Cx46 gap junctional channel exhibits a large asymmetry in voltage sensitivity, we analyzed the gating kinetics at both +V_j_ and –V_j_ when Cx46 cells were held at constant voltage. For both the positive and negative junctional potentials, the gating kinetics for the heterotypic Cx43/Cx46 gap junctional channel also required two exponential components with values of 0.29±0.03 s and 3.6±0.3 s (n=6) for –V_j_, and 0.81±0.08 s and 5.1±0.9 s for +V_j_. On the other hand, the heteromeric gap junction formed by co-expressing Cx43 and Cx46 (i.e., Cx43•46/Cx43•46) exhibited significantly slower gating kinetics, requiring a second-order exponential function with time constants of 1.2±0.3 s and 8.1±2.3 s (n=10).

### Connexin expression in oocyte membranes

**Figure 7:**
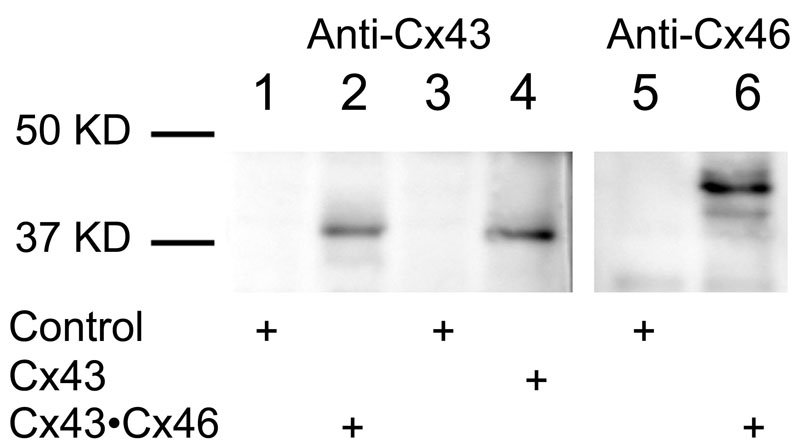
Western blot analysis of Cxs expressed in *Xenopus* oocytes. Lanes 1, 3, 5: uninjected oocytes to serve as non-expressing control. Lanes 2 and 6: oocytes co-expressing Cx43 and Cx46 (Cx43•46). Lane 4: oocytes expressing Cx43 alone. Lanes 1–6 were probed with anti-Cx43 antibody, whereas lanes 5 and 6 were probed with anti-Cx46 antibody.

The expression of Cx subunits in oocyte membranes was probed using western blot analysis. As shown in Figure 7, no Cx specific signals were detected from samples prepared from non-expressing oocytes (lanes 1, 3, 5). For oocytes co-expressing Cx43 and Cx46 (Cx43•46), a Cx43 specific band was detected with a molecular mass of about 43 kDa when probed with anti-Cx43 antibody (lane 2), and a Cx46 band was detected with a molecular mass of about 46 kDa when probed with anti-Cx46 antibody (lane 6). For oocytes expressing Cx43 alone, a band at the appropriate molecular mass of ~43 kDa was also detected in the membrane preparation of the sample (lane 4). These results indicate that the inability to detect hemichannel currents in oocytes expressing Cx43 is not due to a lack of protein synthesis of the subunit in the membrane, but most likely reflects the unique gating property of the Cx43 connexon.

## Discussion

Biochemical [15], microarray [28], and immunocytochemial analyses [14,29] provide conclusive evidence that Cx43 and Cx46 are present and form patent intercellular pathways in RPE cells, and when expressed in cell lines, Cx43 and Cx46 are capable of forming heteromeric complexes [17,18]. Moreover, the junctional permeability between cells expressing these Cxs has been demonstrated by dye coupling studies using molecular tracers [2,30,31]. In the present study, we used the *Xenopus* oocyte expression system to analyze the electrophysiological characteristics of the hemichannels and gap junctional channels that were formed by different combinations of the Cxs expressed in RPE cells.

Similar to previous reports, cf [26], no significant hemichannel currents were observed in single oocytes expressing Cx43. In contrast, positive voltage steps elicited large outward membrane currents from cells expressing Cx46 (Figure 1), and currents of similar magnitude were recorded from cells co-expressing Cx43 with Cx46 (Figure 2). In addition, the voltage sensitivity (V_o_), obtained from fitting the tail current amplitude with the Boltzmann equation, was also similar for these two types of hemichannels (Table 1). However, when Cx43 was co-expressed with Cx46, both the slope of the voltage activation curve and the activation kinetics of the hemichannel changed significantly (Figure 3 and Figure 4). These findings provide good evidence that Cx43 and Cx46 can co-assemble with each other when expressed in *Xenopus* oocytes. For homomeric Cx46 hemichannels, the gating charge of 1.4 was derived by fitting the amplitudes of the tail current with the Boltzmann equation (Table 1). Co-expressing Cx43 with Cx46 increased the gating charge to a value of 1.6 (Figure 3 and Table 1), suggesting more energy is required to open the voltage gate in the heteromeric hemichannels.

Both Cx43 and Cx46 can readily form homotypic GJ when expressed in *Xenopus* oocytes (Figure 5). The junctional conductances were similar (Table 3) and there is general symmetry of the voltage gating behavior for minus and plus V_j_s, similar to what has been reported by White et al. [16]. On the other hand, the gating kinetics measured at −120 mV revealed significant differences between these two Cxs. Whereas curve fitting to describe the currents for Cx43 GJs required two exponential components, those recorded from Cx46 pairs could be described by a single exponential function (Figure 6).

Gap junction communication could also be mediated by heterotypic gap junctions formed by Cx43 and Cx46 (Figure 5). As previously reported [16], the heterotypic GJs showed smaller G_j_ than homotypic GJs, and displayed a much larger gating asymmetry not predictable from its homotypic counterparts. Since homotypic GJs containing either Cx43 or Cx46 exhibited similar junctional conductances, the much smaller conductance of channels formed by heterotypic GJs (Cx43/Cx46) suggests either that the single channel conductance for the heterotypic GJ channel was less, or that the efficiency in forming heterotypic channels was lower, or both. In addition, the heterotypic GJs exhibited larger values for gating charges than their homotypic counterparts (Table 2), and showed asymmetries in voltage sensitivities resulting in V_o_ values of 92 and 46 for +V_j_ and –V_j_, respectively (Table 2). Interestingly, the gating kinetics of the heterotypic GJ channel also contained two exponential components for both positive and negative junctional voltages with respect to the Cx46 cell, with time constants similar to those derived from homotypic Cx43 GJs (Figure 6).

In paired cells expressing both Cx43 and Cx46, GJs that could potentially be formed include a mixture of homotypic Cx43, homotypic Cx46, heterotypic Cx43/46, or heteromeric (Cx43**•**46/Cx43**•**Cx46). Our results indicate that the kinetic properties of the GJ currents recorded from these cells exhibited features that were distinctly different from the currents of homomeric GJs consisting of Cx43 or Cx46 (Figure 6). Specifically, two exponential components were required to fit the current traces of Cx43**•**46 GJs with longer time constants than those derived from homomeric pairs. Since heterotypic Cx43/Cx46 form much smaller GJs compared with their homotypic counterparts (Table 2), it is likely that heterotypic Cx43/Cx46 contribute only a small fraction of the GJ currents recorded from cell pairs co-expressing Cx43 and Cx46. Therefore, our results are consistent with the notion that the heteromeric Cx43**•**46 GJ is the dominant form in cell pairs co-expressing Cx43 and Cx46. In sum, although our findings suggest that junctional communication can occur via the homotypic, heterotypic, and heteromeric gap junctions formed by Cxs 43 and 46, the question of which type(s) of pairing exist in native RPE cells can only be resolved with further study of cells in their natural environment.
